# Supplementary material for: Gibberellin Oxidase Gene Family in L. chinense: Genome-Wide Identification and Gene Expression Analysis
Source: Int J Mol Sci. 2021 Jul 2;22(13):7167. doi: 10.3390/ijms22137167 (PMC8268368; doi:10.3390/ijms22137167)
Supplement: Supplementary file 1 [file ijms-22-07167-s001.zip › Supple/FigS1.pdf]

	Logo	E-value	Sites	Width
1	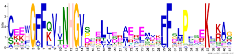	9.9e-411	28	41
2	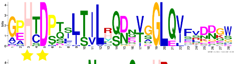	2.3e-349	27	28
3	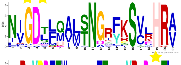	2.6e-339	29	21
4	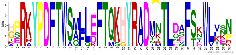	1.0e-176	9	38
5	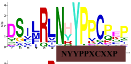	3.1e-167	27	15
6	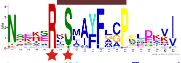	1.1e-185	29	21
7	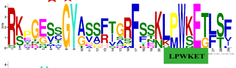	5.6e-140	13	29
8	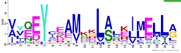	1.1e-138	27	21
9	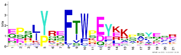	7.5e-110	29	21
10	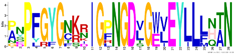	1.3e-094	8	28
11	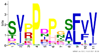	2.3e-062	28	11
12	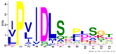	2.7e-054	28	13
13	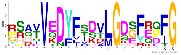	2.8e-030	9	21
14	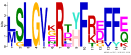	2.1e-028	8	15
15	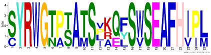	4.2e-028	4	25
