# Supplementary material for: Gibberellin Oxidase Gene Family in L. chinense: Genome-Wide Identification and Gene Expression Analysis
Source: Int J Mol Sci. 2021 Jul 2;22(13):7167. doi: 10.3390/ijms22137167 (PMC8268368; doi:10.3390/ijms22137167)
Supplement: Supplementary file 1 [file ijms-22-07167-s001.zip › Supple/LetPub - Certificate 2021.pdf]

Certificate of English Language Editing

Manuscript Title:

The gibberellin oxidase gene family in Liriodendron chinense: genome-wide identification and expression in response to abiotic stress and phytohormones

Date of Revision:

June 3, 2021

Abstract:

Studying the gibberellin (GA) synthesis pathway can provide a basis for the directional breeding of Liriodendron chinense to meet market demand. However, a systematic analysis of GAox in the angiosperm L. chinense has not yet been reported. Here, we identified all LcGAox gene family members in L. chinense, which were classified into the three subgroups of GA20ox, C19GA2ox, and C20GA2ox. Comparison of the gene structure, conserve motifs, phylogenetic relationships, and syntenic relationships of gibberellin oxidase gene families in different species indicated that the gene functional differences may be due to the partial deletion of their domains during evolution. Furthermore, evidence for purifying selection was detected between orthologous GAox genes in rice, grape, Arabidopsis, and L. chinense. Analysis of the codon usage patterns showed that mutation pressure and natural selection might have induced codon usage bias in angiosperms; however, the LcGAox genes in mosses, lycophytes, and ambarella plants exhibited no obvious codon usage preference. These results suggested that the gibberellin oxidase genes were more primitive. The gene expression pattern was analyzed in different tissues subjected to multiple abiotic stresses, including GA, abscisic acid (ABA), and chlormequat (CCC) treatment, by RNA-seq and qRT-PCR, ...

This document certifies that the manuscript listed above was copy edited for English language by LetPub, with regard to grammar, punctuation, spelling, and clarity. All of our language editors are native English speakers with long-term experience in editing scientific and technical manuscripts. We are committed to leveling the playing field for researchers whose native language is not English.

- Documents receiving this certification should be regarded as having undergone professional editorial revision for English language before submission. However, the authors may accept or reject LetPub's suggestions and changes at their own discretion and LetPub does not have editorial control over the submitted documents.
- The language quality of the submitted document is the sole responsibility of the submitting authors subject to those authors' adherence to LetPub's revisions and instruction. LetPub's provision of service does not constitute a guarantee or endorsement of the authors' work herein.
- Neither the research content nor the authors' intended meaning were altered in any way during the editing process.
- If you have any questions or concerns about this edited document, please contact us at support@letpub.com

LetPub
Your Manuscript Editor

LetPub is an author service brand owned and operated by Accdon LLC. Headquartered in the Boston area, we are a full-spectrum author services company with a large team of US-based certified language and scientific editors, ISO 17001 accredited translators, and professional scientific illustrators and animators. We advocate ethical publication practices and are an official member of the Committee on Publication Ethics (COPE).

For more information about our company, services, and partnership programs, please visit www.letpub.com.

© 2021 Accdon, LLC. All Rights Reserved. Tel: 1-781-202-9968 Email: info@accdon.com Address: 400 Fifth Ave, Suite 530, Waltham, MA 02451, United States
