# Supplementary figures and images for: Gibberellin Oxidase Gene Family in L. chinense: Genome-Wide Identification and Gene Expression Analysis
Source: Int J Mol Sci. 2021 Jul 2;22(13):7167. doi: 10.3390/ijms22137167 (PMC8268368; doi:10.3390/ijms22137167)

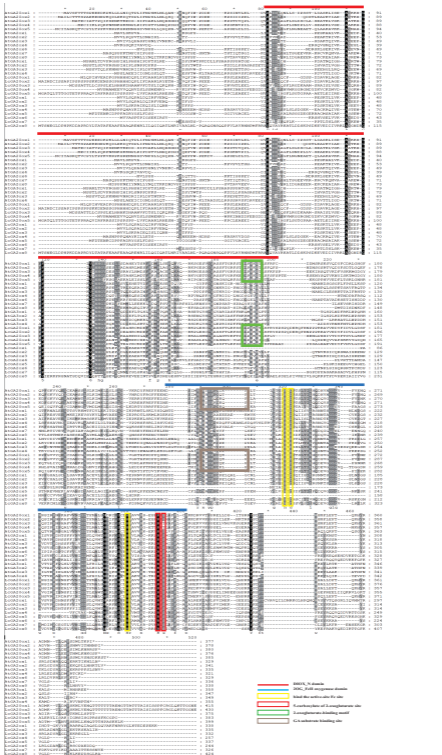

Supplement: Supplementary file 1 [file ijms-22-07167-s001.zip › Supple/FigS2.pdf]

**GA20**

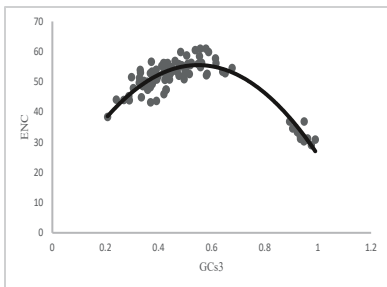

**C19GA2**

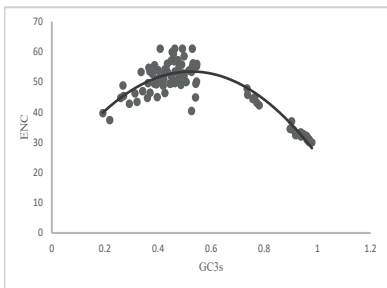

**C20GA2**

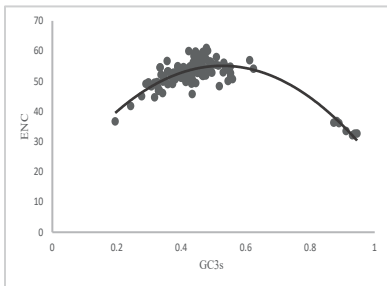

Supplement: Supplementary file 1 [file ijms-22-07167-s001.zip › Supple/FigS3.pdf]
